# Quantifying the advantages of conducting a prospective meta-analysis (PMA): a case study of early childhood obesity prevention

**DOI:** 10.1186/s13063-020-04984-x

**Published:** 2021-01-22

**Authors:** A. L. Seidler, K. E. Hunter, D. Espinoza, S. Mihrshahi, L. M. Askie, on behalf of the EPOCH Collaboration, Lisa M. Askie, David Espinoza, Andrew Martin, Lynne A. Daniels, Seema Mihrshahi, Rachael Taylor, Li Ming Wen, Karen Campbell, Kylie D. Hesketh, Chris Rissel, Barry Taylor, Anthea Magarey, Anna Lene Seidler, Kylie E. Hunter, Louise A. Baur

**Affiliations:** 1 NHMRC Clinical Trials Centre, University of Sydney, Sydney, Australia; 2 NHMRC Centre of Research Excellence in the Early Prevention of Obesity in Childhood, Prevention Research Collaboration, Sydney School of Public Health, University of Sydney, Camperdown, NSW Australia; 3 Department of Health Systems and Populations, Faculty of Medicine, Health and Human Sciences, Macquarie University, Sydney, NSW 2109 Australia

**Keywords:** Prospective meta-analysis, Outcome harmonisation, Systematic reviews, Methodology, Collaboration, Early childhood obesity prevention, Individual participant data

## Abstract

**Background:**

For prospective meta-analyses (PMAs), eligible studies are identified, and the PMA hypotheses, selection criteria, and analysis methods are pre-specified *before* the results of any of the studies are known. This reduces publication bias and selective outcome reporting and provides a unique opportunity for outcome standardisation/harmonisation. We conducted a world-first PMA of four trials investigating interventions to prevent early childhood obesity. The aims of this study were to quantitatively analyse the effects of prospective planning on variations across trials, outcome harmonisation, and the power to detect intervention effects, and to derive recommendations for future PMA.

**Methods:**

We examined intervention design, participant characteristics, and outcomes collected across the four trials included in the EPOCH PMA using their registration records, protocol publications, and variable lists. The outcomes that trials planned to collect prior to inclusion in the PMA were compared to the outcomes that trials collected after PMA inclusion. We analysed the proportion of matching outcome definitions across trials, the number of outcomes per trial, and how collaboration increased the statistical power to detect intervention effects.

**Results:**

The included trials varied in intervention design and participants, this improved external validity and the ability to perform subgroup analyses for the meta-analysis. While individual trials had limited power to detect the main intervention effect (BMI *z*-score), synthesising data substantially increased statistical power. Prospective planning led to an increase in the number of collected outcome categories (e.g. weight, child’s diet, sleep), and greater outcome harmonisation. Prior to PMA inclusion, only 18% of outcome categories were included in all trials. After PMA inclusion, this increased to 91% of outcome categories. However, while trials mostly collected the same outcome categories after PMA inclusion, some inconsistencies in how the outcomes were measured remained (such as measuring physical activity by hours of outside play versus using an activity monitor).

**Conclusion:**

Prospective planning led to greater outcome harmonisation and greater power to detect intervention effects, while maintaining acceptable variation in trial designs and populations, which improved external validity. Recommendations for future PMA include more detailed harmonisation of outcome measures and careful pre-specification of analyses to avoid research waste by unnecessary over-collection of data.

## Background

Systematic reviews with and without meta-analyses are widely used to inform health care guidelines, policies, and medical practice [1, 2]. However, there are several limitations and potential sources of bias associated with using these traditional aggregate data approaches to synthesise evidence: the need to extract data from existing publications and other sources limits the availability of outcome data that is suitable for combined analysis [3], the knowledge of study results prior to conducting a systematic review may result in a data-dependent selection of subgroups or outcomes [4], and there is the risk of publication bias and selective outcome reporting, since positive findings are more likely to be published and reported and thus identified for inclusion in the systematic review [5, 6].

Prospective meta-analysis (PMA) is an approach that addresses many of these concerns and the potential sources of bias associated with traditional retrospective meta-analysis [7, 8]. For a PMA, eligible studies are identified and the PMA hypotheses, selection criteria, and analysis methods are specified before any results of the included studies related to the PMA research question are known [8]. Therefore, hypotheses, selection criteria, and outcomes for the PMA cannot be influenced by the results of the individual studies (since they are specified before the results are known), and publication bias and selective outcome reporting are reduced. A further advantage of including studies in their planning stages is the potential to harmonise outcome collection: studies can agree to collect the same outcomes, using the same measures. This can greatly improve the number of common outcomes included in the resulting meta-analysis. A PMA will also have more power than individual included studies to detect smaller treatment effects. Studies in a PMA can thus agree to collect and analyse additional uncommon outcomes (for instance, rare but serious side effects) that they would not have had the power to detect individually [9]. Despite the harmonisation of key outcomes, PMAs allow more variation in individual studies than multi-centre studies with respect to the choice of participants, intervention design, and focus of the individual studies [10]. This increases the external validity of PMA, while maintaining the benefits of attaining a complete combined dataset with key outcomes available for all included studies [9]. All these benefits of PMA have been outlined by multiple sources [3, 4, 8–12], yet to date, they have not been analysed quantitatively.

In this study, we quantified the advantages of PMA using the Early Prevention of Obesity in Children (EPOCH) PMA as an example. The EPOCH PMA was a world-first individual participant data PMA of four Australasian randomised controlled trials that investigated interventions to prevent early childhood obesity in a total of 2196 mother-child dyads [13–18]. This was an important study since in Australia, more than 1.2 million children over 2 years of age are living with overweight or obesity [19]. Eligible trials were identified by searching trial registries, MEDLINE and EMBASE, and approaching relevant paediatric networks and participants/speakers at conferences (further information has been published elsewhere) [17]. All trials tested the effectiveness of obesity prevention interventions providing anticipatory guidance on feeding and activity behaviours to first-time parents. All included interventions commenced in infancy (child < 6 months) and had an assessment of outcomes at 18–24 months of age. The PMA looked at changes in BMI *z*-score as the primary outcome (finding a statistically significant difference of − 0.12) and analysed a range of secondary outcomes and subgroups. The trials were identified for inclusion, hypotheses were agreed, and all analyses were planned and published in a protocol [17], before any of the individual trial results were known. The trials forming the EPOCH Collaboration were all planned independently and prospectively registered before learning about each other and deciding to collaborate in a PMA. This provided a unique opportunity to assess how the decision to collaborate in a PMA changed the study design compared to the original registration records.

The aims of this study were as follows:

1. To analyse the effects of prospectively planning a meta-analysis for the EPOCH PMA, by examining:
  - The variation in the intervention design and participants across trials
  - How inclusion in the PMA affected outcome harmonisation
  - How inclusion in the PMA changed the statistical power to detect intervention effect differences between the intervention and control group by increasing the available sample size
2. To derive recommendations for future PMAs

## Methods

### Study design and eligible studies

This was a multi-method study, including the four randomised controlled trials that comprised the EPOCH PMA [18]. Three of the included trials, NOURISH [14, 20], Healthy Beginnings Trial (HBT) [13, 21], and Infant Feeding Activity and Nutrition Trial (InFANT) [15, 22], were conducted in Australia, while the Prevention of Overweight in Infancy (POI.nz) [16, 23] trial was conducted in New Zealand.

The EPOCH Collaboration was officially formed in 2009, it was prospectively registered (ACTRN12610000789066), and a joint protocol was published in 2010 [17]. While for some trials recruitment and interventions had already started at this point, outcomes to be measured at the 18-month/2-year follow-up could still be harmonised across all trials. This was achieved by sharing all case report forms, and via discussion at regular collaborator teleconferences and meetings.

### Data sources

#### Registration records

The trials all obtained funding independently and were prospectively registered on ANZCTR or ClinicalTrials.gov between March 2007 and May 2009, prior to joining the EPOCH Collaboration (registration numbers: NCT00892983, ISRCTN81847050, ACTRN12607000168459, ACTRN12608000056392). The original registration records thus provide information on study characteristics and planned outcomes *prior to inclusion in the PMA*.

#### Variable map

Upon completion, all trials provided complete de-identified individual participant data for inclusion in the combined EPOCH meta-analysis to the central data coordination centre. The data provided were summarised in a variable map, indicating which variables were available for which trials at which time points, and how they were measured. The variable map provided information on outcomes the trials collected *after inclusion in the PMA*.

#### Trial protocols and results papers

All of the individual trials published protocols and results papers [13–16, 20–23] outlining the characteristics of their specific interventions such as content and setting. These materials were used to extract information on study characteristics.

### Measures

#### Trial characteristics

Characteristics of the four included trials were extracted from the trial protocols, intervention materials, and main outcome papers. Trial characteristics assessed for this study include the content covered in the intervention, age of infants at intervention start, intervention procedures, facilitator type, session length and frequency (dose), and delivery mode (individual or home).

#### Outcome categories

Outcome category was defined as the broad construct researchers were interested in assessing. Examples of outcome categories as defined for this analysis are as follows: weight, child’s diet, breastfeeding, and sleep. Outcome categories *before* inclusion in the PMA were measured by extracting all the outcomes the trial investigators stated they planned to collect in the prospective registration records, which were then coded into outcome categories. Outcome categories *after* inclusion in the PMA were measured by coding all outcomes listed in the variable map into the available outcome categories or new categories if necessary. The coding process was iterative; the outcome categories were coded by one author and then discussed with and refined by a second author.

#### Outcome measurements

The specific outcome measure (e.g. specific questionnaire items) assessed within each outcome category was extracted from the variable map. This information was only available for the time point *after* inclusion in the PMA, since registration records did not necessarily include this level of detail.

### Analyses

Trial characteristics were examined and compared to detect variations across trials. To analyse outcome harmonisation, the outcome categories collected by each trial were compared before and after inclusion in the PMA. For this purpose, the proportion of matching outcome categories across trials was computed prior to, and post, PMA inclusion, and the number of outcomes and rates of harmonisation were compared across and within trials. Furthermore, the specific outcome measures were analysed and compared across trials. The power for detecting the observed difference in the primary outcome of BMI *z*-score of − 0.12 in an intention-to-treat analysis was calculated for the four individual trials separately, and for the combined PMA, using the software g-power [24].

## Results

The characteristics of the included trials are shown in Table 1. While all four trials were offering early interventions aimed at preventing early childhood obesity that were delivered at least in part face-to-face, there was some variation in participants and intervention design across trials. Two of the trials started before the baby was born (antenatally), while the other two started in early infancy. One trial was delivered in individual sessions via home visits, two were delivered in a group setting, and one trial combined home visits with group sessions. In addition, there was some variation in intervention content and procedures. These differences across trials allowed for subgroup analyses in the EPOCH PMA [18] and for additional qualitative analyses comparing experiences of investigators and facilitators across trials [25].

**Table 1:** Intervention characteristics of trials included in EPOCH Collaboration

	Healthy Beginnings Trial	InFANT	NOURISH	POI.nz
**Number of participants**	*N* = 667 first-time mothers	*N* = 698 first-time mothers	*N* = 559 first-time mothers	*N* = 400 first-time mothers
**Intervention content**	Promote/sustain breastfeeding Bottle feeding advice Introduction of solids Amount or feeding frequency Limit SSB Limit certain foods (e.g. sweets) Response hunger/satiety cues Promote tummy time Promote play or activity Limit TV/screen time	Introduction of solids Amount or feeding frequency Limit SSB Limit certain foods (e.g. sweets) Repeat food exposure Response hunger/satiety cues Fussy eating Promote tummy time Promote play or activity Limit TV/screen time	Bottle feeding advice Introduction of solids Amount or feeding frequency Limit SSB Limit certain foods (e.g. sweets) Repeat food exposure Response hunger/satiety cues Fussy eating Promote tummy time Promote play or activity Limit TV/screen time	Promote/sustain breastfeeding Bottle feeding advice Introduction of solids Amount or feeding frequency Limit SSB Limit certain foods (e.g. sweets) Repeat food exposure Response hunger/satiety cues Fussy eating Promote tummy time Promote play or activity Limit TV/screen time Sleep timing Sleep environment
**Intervention start**	Antenatal	Infant aged 4–6 months	Infant aged 3 months	Antenatal
**Procedures**	Participant-led discussion guided by checklist	Brief didactic sessions Group discussion Peer support Exploration of perceived barriers	Didactic sessions Group discussion Peer support Monitoring/discussion of progress at home	Face-to-face individual sessions Group physical activity sessions
**Intervention facilitator**	Community nurse	Dietitian	Dietitian and psychologist	Trained researchers and lactation consultants
**Delivery mode**	Individual: home visits	Group: educational peer support modules at maternal and child health centres	Group: sessions delivered to pre-existing mothers groups at child health services	Individual and group: at home, a clinic, and a community hub
**Intervention dose**	24 months 8 sessions	15 months 6 sessions	12 months 12 sessions	18 months 2–10 sessions

This information has been extracted from Askie et al. [17], Seidler et al. [25], and the individual trial protocols and manuscripts [13–16, 20–23]

*SSB* sugar-sweetened beverages

**Fig. 1:**
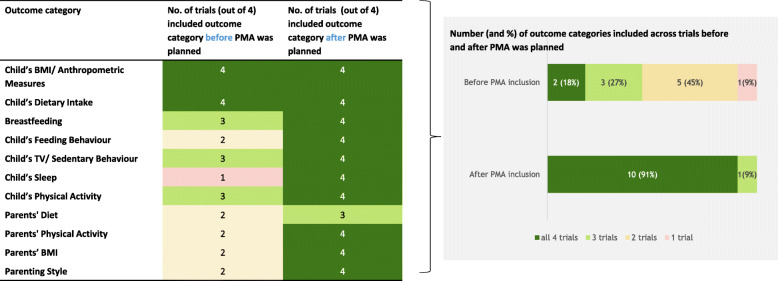
Main outcome categories and numbers of trials including them before and after prospective meta-analysis was planned

As shown in Fig. 1, the outcomes the trials planned to collect prior to inclusion in the PMA (as specified in the registry records) were coded into eleven outcome categories. No new outcome categories were identified when examining the outcomes collected after inclusion in the PMA, but the existing outcome categories were covered by a greater number of trials. Before PMA inclusion, only 18% (*n* = 2) of the outcome categories were intended to be collected by all four trials, and 54% (*n* = 6) of the outcome categories were included in only one or two trials. After PMA inclusion, all outcome categories were measured by at least three trials; one outcome category was measured by three of the four trials, and all other outcome categories (*n* = 10, 91%) were collected by all trials. The numbers of outcome categories collected in the individual trials increased by 54%, from an average of seven to an average of eleven outcome categories per trial.

However, as shown in Table 2, the specific measures and tools used to assess each outcome category varied substantially across trials. Only 7% of questions and measures were identical across all four trials, while 59% of all specific outcome measures were only used by one of the four trials.

**Table 2:** Overlap across trials in exact measures and tools used to assess outcomes after prospective meta-analysis was planned

Measures and tools …	Number	Per cent
… included by all 4 trials	17	7
… included by 2–3 of the trials	89	34
… included by only 1 trial	153	59

Combining data from the four trials increased the sample size and therefore the statistical power to detect the observed mean difference of − 0.12 found for the primary outcome, BMI *z*-score [18]. As shown in Table 3, the individual trials had minimal power (all less than 0.35) to detect this intervention effect (they were instead conceptualised and powered to detect differences in their respective primary outcomes) which was also reflected in only one of the individual four trials finding a statistically significant intervention effect for BMI *z*-score. The combined analysis of all trials, however, had a power of 0.83 to detect an effect of this size for the primary outcome.

**Table 3:** Power to detect a − 0.12 mean difference in BMI *z*-scores for the individual trials and the EPOCH PMA

Trial	No. of participants	Power to detect observed mean difference of − 0.12
Healthy Beginnings	667	0.34
InFANT	698	0.29
NOURISH	542	0.35
POI.nz	289	0.16
**Combined to EPOCH PMA**	**2196**	**0.83**

Note that each of the included trials was individually powered to detect differences in their respective primary outcomes

## Discussion

The included trials varied in intervention design and participant characteristics, which improved external validity and the ability to perform subgroup analyses. The decision to collaborate in a PMA led to greater outcome harmonisation with 18% of outcome categories included in all trials prior to PMA inclusion versus 91% after PMA inclusion, and a 54% increase in the number of collected outcome categories. However, on a more detailed level when assessing specific outcome measures, only 7% of measures were identical across all trials. While individual trials had limited power to detect the observed intervention effect for the primary outcome, combining their data substantially increased the statistical power.

### Strengths and limitations

To our knowledge, this study is the first to quantify the advantages of a PMA in increasing data harmonisation. We made use of a range of data sources, and the extensive records documenting the planning and conduct of the individual studies and the PMA. The availability of registry records that recorded which outcomes the trials had planned to collect prior to inclusion in the PMA enabled a comparison of outcomes prior to, and post, PMA inclusion.

The main limitation to this study is that the registry records that were used to measure outcome categories before PMA inclusion were less detailed than the variable maps used to measure outcome categories after PMA inclusion. This difference in the level of detail provides an alternative explanation for the greater extent of outcome harmonisation and the increase in collected outcome categories. Potentially, trials did not record all outcomes they planned on collecting in their registry records. However, there are three reasons why this is an unlikely sole explanation for the large observed increase in outcome harmonisation. Firstly, we used broad outcome categories to quantify outcomes to account for less detail in the registration records. Secondly, a major aim of prospective trial registries is to record all outcomes that trials plan to collect [26], and most trial registries permit large numbers of outcomes to be recorded [27]. Thirdly, no new outcome categories needed to be created to code the outcomes the trials collected after inclusion in the PMA. That is, all additional outcomes the trials collected after inclusion in the PMA fitted into the pre-existing outcome categories derived from the registration records of at least one of the other trials. This suggests that the observed additional outcome categories were collected in response to outcome categories collected by other trials in an effort to increase outcome harmonisation and are not artefacts of different levels of detail.

### Interpretation of findings

One of the main differences between PMA and multi-centre trials is that in a PMA, individual participating sites have greater autonomy [8, 9]. Trials in a PMA aim for a high level of data *harmonisation*, without complete outcome *standardisation*, across all trials as would occur in a multi-centre trial. In the PMA used in this study, this greater independence resulted in substantial variability between intervention design, timelines, and participant groups across trials. This has the advantage of a heightened external validity, with results being more generalisable as they are not restricted to one particular centre, intervention, or population group.

While this variability in trial design is desirable to some extent, outcome harmonisation in a PMA ensures the ability to conduct meaningful combined analyses [8]. Our results clearly show how outcome harmonisation improved after the decision to collaborate in a PMA was made, with outcome category harmonisation increasing from 18 to 91% of outcome categories being collected by all trials. This increase in harmonisation led to an increase in the amount of data that were collected by each trial, resulting in slightly increased resources required for data collection by the individual trials than they had originally planned. Yet, the resulting increase in total combined data availability enabled us to answer many more research questions than would have been possible without the PMA data harmonisation process.

The increase in statistical power to detect treatment effects constitutes one of the main advantages and reasons for synthesising evidence. Increasing sample size strengthens the chance of detecting effects, and it enables us to determine the size of these effects with greater certainty [28]. Increased outcome harmonisation directly translates into more outcomes being available for combined analyses and thus greater power to detect potential treatment effect differences.

**Fig. 2:**
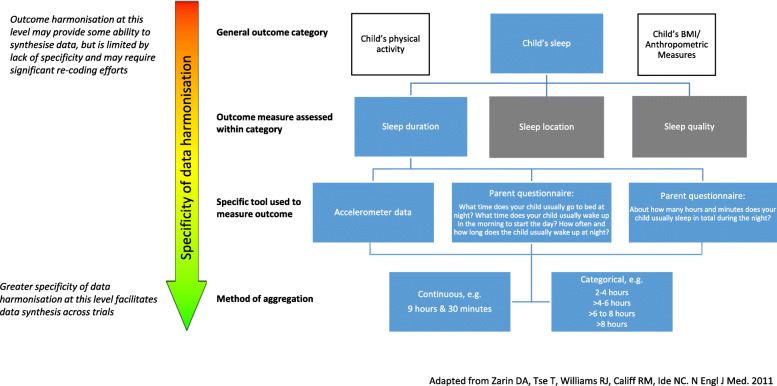
Outcome measures framework, using child’s sleep duration as an example. Adapted from Zarin et al. [29]

However, while we succeeded in improving outcome category harmonisation across trials, there were still residual differences in how these outcomes were operationalised, reflecting a problem in the specificity of the data harmonisation process. When looking at the outcome measures assessed within outcome categories, only 7% were identical in all trials. Figure 2 shows the different levels of specificity that can be used to describe outcomes, and the importance of a high level of specificity for outcome harmonisation. For some outcomes, the way they were to be measured was not pre-specified in sufficient detail. This led to trials choosing different measures or tools for the same outcome category, and the data managers at the central data collection centre had to find ways of converting these measures to common outcome variables. For example, some trials assessed sleep duration by asking ‘What time does your child usually go to bed at night?’, ‘What time does your child usually wake up in the morning to start the day?’, and ‘How often and how long the child usually wakes up at night?’, while other trials simply asked ‘About how many hours and minutes does your child usually sleep in total during the night?’. While these different measures can both be used to derive the same outcome of ‘sleep duration’, there was a significant computational effort associated with the derivation, and it is possible that the trial which took into account ‘waking up at night’ time systematically led to lower total sleep duration estimates, and potentially unintended increased heterogeneity.

Yet, in a PMA, it is not expected nor desirable to have 100% harmonisation across outcome measures. One reason for this is that within a PMA, trials can have additional focus areas that are only covered by an individual trial, and do not have to be assessed by all trials—this is a major desirable feature and stands in contrast to multi-centre trials in which all trials usually collect the same information. For instance, in the EPOCH PMA, the POI.nz trial had a particular focus on sleep, and collected over 50 additional variables related to sleep, including the resource-intensive use of an accelerometer. It was not desirable nor feasible for all trials to collect these extra outcome measures, but instead, all the other EPOCH trials added a few common core measures of the outcome category sleep to their data collection forms.

### Recommendations for future PMAs

While increased outcome harmonisation enables greater data synthesis and improved statistical power to detect intervention effects, this needs to be balanced against an unnecessary collection of data if this leads to an undue burden on participants and research waste. For future PMAs, we recommend careful consideration and extensive dialogue about the amount of core common data that is necessary and desirable to answer all relevant research questions as early as possible in the planning phase of both the PMA and the participating individual trials. If already existing, agreed core datasets [30] within particular specialities should be the basis for these decisions.

To avoid differences in how common outcomes are measured and operationalised across trials, we recommend that future PMA collaborations be more specific a priori regarding how they plan to measure common outcomes at different levels, as displayed in Fig. 2. For example, for the outcome category ‘breastfeeding’, the measurement tool may be a self-reported questionnaire asking ‘Has your child ever been breastfed?’ To ensure all trials collect this measure consistently, the outcome would need to be defined very explicitly. In this case, ‘ever been breastfed’ may be defined as the infant having received breast milk even just once, including putting the infant to the breast to feed or giving expressed breast milk. Consistent definitions and measurement methods for common outcomes greatly enhance the ability to synthesise data and reduce the amount of recoding and cleaning necessary to achieve this. Each trial is nonetheless able to collect additional trial-specific outcomes for their own purposes.

## Conclusion

This study quantifies the advantages of conducting a PMA: prospective planning led to greater outcome harmonisation, and greater power to detect treatment effects, while maintaining variation in trial designs and studied populations which heightens external validity. PMAs therefore combine the benefits of meta-analyses and multi-centre trials. Future PMAs should be more specific in their data harmonisation processes, pre-specifying these as early as possible in the PMA planning phase, to maximise the possible combined analyses while avoiding unnecessary over-collection of data. The EPOCH PMA is an example of how collaborations can answer important research questions with higher certainty while minimising bias. The lessons learnt from this PMA can help researchers to successfully collaborate in similar future projects while minimising research waste.

## Data Availability

The datasets used and/or analysed during the current study are available from the corresponding author on reasonable request and with the permission of the members of the EPOCH Collaboration.

## Abbreviations

ANZCTR: Australian New Zealand Clinical Trials Registry
BMI: Body mass index
EPOCH: Early Prevention of Obesity in Children
HBT: Healthy Beginnings Trial
InFANT: Infant Feeding Activity and Nutrition Trial
NHMRC: National Health and Medical Research Council
PMA: Prospective meta-analysis
POI.nz: Prevention of Overweight in Infancy
